# C5aR1 Activation Drives Early IFN-γ Production to Control Experimental *Toxoplasma gondii* Infection

**DOI:** 10.3389/fimmu.2020.01397

**Published:** 2020-07-08

**Authors:** Daria Briukhovetska, Birte Ohm, Fabian T. Mey, Julio Aliberti, Marie Kleingarn, Markus Huber-Lang, Christian M. Karsten, Jörg Köhl

**Affiliations:** ^1^Institute for Systemic Inflammation Research, University of Lübeck, Lübeck, Germany; ^2^Division of Immunobiology, Cincinnati Children's Hospital and College of Medicine, University of Cincinnati, Cincinnati, OH, United States; ^3^Institute of Clinical and Experimental Trauma-Immunology, University Hospital of Ulm, Ulm, Germany

**Keywords:** complement, C5a, C5a receptor 1, *Toxoplasma gondii*, dendritic cell, NK cell, interferon-gamma, interleukin-12

## Abstract

*Toxoplasma gondii* (*T. gondii*) is a parasite infecting animals and humans. In intermediate hosts, such as humans or rodents, rapidly replicating tachyzoites drive vigorous innate and adaptive immune responses resulting in bradyzoites that survive within tissue cysts. Activation of the innate immune system is critical during the early phase of infection to limit pathogen growth and to instruct parasite-specific adaptive immunity. In rodents, dendritic cells (DCs) sense *T. gondii* through TLR11/12, leading to IL-12 production, which activates NK cells to produce IFN-γ as an essential mechanism for early parasite control. Further, C3 can bind to *T. gondii* resulting in limited complement activation. Here, we determined the role of C5a/C5aR1 axis activation for the early innate immune response in a mouse model of peritoneal *T. gondii* infection. We found that *C5ar1*^−/−^ animals suffered from significantly higher weight loss, disease severity, mortality, and parasite burden in the brain than wild type control animals. Severe infection in *C5ar1*^−/−^ mice was associated with diminished serum concentrations of IL-12, IL-27, and IFN-γ. Importantly, the serum levels of pro-inflammatory cytokines, including IL-1α, IL-6, and TNF-α, as well as several CXC and CC chemokines, were decreased in comparison to wt animals, whereas anti-inflammatory IL-10 was elevated. The defect in IFN-γ production was associated with diminished *Ifng* mRNA expression in the spleen and the brain, reduced frequency of IFN-γ^+^ NK cells in the spleen, and decreased *Nos2* expression in the brain of *C5ar1*^−/−^ mice. Mechanistically, DCs from the spleen of *C5ar1*^−/−^ mice produced significantly less IL-12 in response to soluble tachyzoite antigen (STAg) stimulation *in vivo* and *in vitro*. Our findings suggest a model in which the C5a/C5aR1 axis promotes IL-12 induction in splenic DCs that is critical for IFN-γ production from NK cells and subsequent iNOS expression in the brain as a critical mechanism to control acute *T. gondii* infection.

## Introduction

*T. gondii* is an obligate intracellular apicomplexan parasite capable of infecting virtually all nucleated animal cells (1). Typically, infection occurs after ingestion of oocysts or tissue cysts that release the fast-replicating form of the parasite—the tachyzoites, which multiply asexually and spread through the host [reviewed in (2)]. Following successful immune system activation, the parasite converts into the slowly replicating bradyzoites and persists asymptomatically as a latent infection in the central nervous system or muscle tissue in the form of cysts (2).

As an intracellular parasite, *T. gondii* drives the induction of typical Th1 immune responses, in which IL-12 and IFN-γ are indispensable to control the infection (3). In mice, immune recognition of *T. gondii* occurs through binding of profilin to the intracellular toll-like receptor (TLR) 11/12 dimers in CD8α^+^ splenic DCs, which act as a primary source for IL-12 (4–8). Secreted IL-12, together with TNFα and IL-18, stimulate NK and T cells to produce IFN-γ (9–11). Such IFN-γ primes infected cells to express immunity-related GTPases (IRGs) and inducible nitric oxide synthase (iNOS, NOS2) as important effector agents [reviewed in (12)]. Reactive nitrogen species synthesized by iNOS exert microbicidal activity in macrophages and microglia and are also essential for the control of *T. gondii* in the brain during the chronic stage of infection (13).

In addition to the cellular immune system, the complement system functions as an essential arm of innate humoral immunity. Studies performed in the '80s have shown that the central complement component C3 can bind to *T. gondii* tachyzoites and activate the alternative pathway (AP). However, alternative pathway activation was shown to be inefficient and did not lead to parasite lysis. Additional activation of the classical pathway (CP) increased the formation of the membrane attack complex (MAC) and the sensitivity of the parasite to complement-mediated killing (14, 15). A recent report identified the lectin pathway (LP) in addition to AP activation in *T. gondii* type I and II strains (16). The authors further showed that *T. gondii* binds the CP and LP regulator C4 binding protein (C4BP) and the AP regulator Factor H (FH). These complement regulators inactivated C3b and limited the formation of the MAC. On the other hand, they found that C3-deficient mice were more susceptible to i.p. *T. gondii* infection resulting in higher mortality than in wt control mice due to uncontrolled parasite proliferation in several tissues. These findings demonstrate a host protective role for pathways downstream of C3. C3 is cleaved into the anaphylatoxin (AT) C3a and the opsonin C3b. C3a exerts its effector functions through activation of its cognate G protein-coupled C3a receptor (C3aR) (17) expressed on several immune cells (18). C3b and its degradation products can either activate immune cells directly through binding to complement receptors type 1 (CR1), CR2 or CR3 (19) or serve as the nucleus to form C5 convertases that cleave C5 into C5a and C5b. C5a is the second AT that exerts its pleiotropic functions through binding and activation of its cognate G protein-coupled C5a receptor 1 (C5aR1, CD88) (20), and C5a receptor 2 (C5aR2, C5L2, GPR77) (21) both of which are mainly expressed by immune cells of the myeloid lineage (18, 22, 23). Several reports have shown that C5aR1 signaling pathways intersect with TLR signaling in many ways, thereby modulating TLR-driven immune responses critical for immune sensing and resistance against several pathogens [reviewed in (24, 25)]. In this context, C5a/C5aR1 axis controls the development of Th1 immune responses in response to several viruses and intracellular parasites (26, 27) through autocrine, TLR-driven AT generation in DCs (28, 29) and subsequent C5aR1 activation resulting in DC cytokine production (29). Importantly, in the absence of C3aR1 and C5aR1, mouse splenic cells failed to produce IL-12 and IFN-γ in response to STAg (26). Our previous findings suggest that CD8α^+^ splenic DCs express C5aR1, but neither C3aR nor C5aR2 [reviewed in (30)].

Based on these findings, we hypothesized that C5a generation during the early *T. gondii* infection and consecutive activation of C5aR1 on splenic DCs are critical for early protective innate immunity. To test this hypothesis, we infected C57BL/6 wt and *C5ar1*^−/−^ mice i.p. with cysts of the *T. gondii* type II strain ME49 and determined complement activation, susceptibility to infection, and parasite burden. We also assessed the production of several pro- and anti-inflammatory cytokines *in vivo* and *in vitro* with a particular emphasis on IL-12 family cytokines and IFN-γ induction as these cytokines play crucial roles in *T. gondii* control. Here, we identified a critical role for C5a/C5aR1 axis activation in CD8α^+^ splenic DCs resulting in IL-12 and subsequent IFN-γ production from NK cells during the first week after infection, eventually controlling parasite proliferation and persistence.

## Materials and Methods

### Reagents

Monoclonal antibodies: PE anti-mouse IL-12/23 (p40; clone C15.6, BD Biosciences Cat# 554479, RRID:AB_395420), Brilliant Violet 510™ anti-mouse/human CD11b (clone M1/70, BioLegend Cat# 101245, RRID:AB_2561390), Brilliant Violet 421™ anti-mouse CD8a (clone 53-6.7, BioLegend Cat# 100737, RRID:AB_10897101), Alexa Fluor® 700 anti-mouse NK-1.1 (clone PK136, BioLegend Cat# 108729, RRID:AB_2074426), PE anti-mouse IFN-γ, eBioscience (clone XMG1.2, Thermo Fisher Scientific Cat# 12-7311-82, RRID:AB_466193), APC anti-mouse CD11c, eBioscience (clone N418, Thermo Fisher Scientific Cat# 17-0114-82, RRID:AB_469346), PE anti-mouse CD11c (clone HL3, BD Biosciences Cat#561044, RRID:AB_2033996), PerCP-Cy5.5 anti-mouse CD3e, eBioscience (clone 145-2C11, Thermo Fisher Scientific Cat# 45-0031-82, RRID:AB_1107000), eFluor450 anti-mouse CD3 (clone 17A2, Thermo Fisher Scientific Cat# 48-0032-82, RRID:AB_1272193), eFluor450 anti-mouse CD19 (clone eBio1D3, Thermo Fisher Scientific Cat# 48-0193-80, RRID:AB_2637304), APC-Cy7 anti-mouse Ly6G (clone 1A8, BioLegend Cat# 127623, RRID:AB_10645331). Cytofix/Cytoperm was from BD Biosciences. Red blood cell (RBC) lysis buffer was prepared using 155 mM NH^4^Cl, 10 mM KHCO_3_, and 0.1 mM EDTA (all from Sigma-Aldrich, Merck). DNase I (Fermentas) and the RevertAid First Strand cDNA Synthesis Kit for mRNA generation was from Thermo Fisher Scientific. Primers for real-time RT-PCR were from Eurofins Genomics, and all other reagents for RT-PCR were from Bio-Rad. RPMI 1640 medium, Dulbecco's PBS (DPBS), L-glutamine, penicillin, and streptomycin were from Life Technologies. BSA was from Sigma-Aldrich, Merck.

### Mice

Wild type C57BL/6 mice were purchased from Janvier Labs (Le Genest Saint Isle, France). *C5ar1*^−/−^ (B6.129S4-*C5ar1*^*tm*1*Cge*^, MGI:6382541) mice (31) on the C57BL/6 background were bred and housed under specific pathogen-free conditions in the animal facilities of the University of Lübeck, Germany, and Cincinnati Children's Hospital Medical Center, Cincinnati, Ohio, USA. NMRI mice were purchased from Charles River (Sülzfeld, Germany). Animals were used at 8–12 weeks of age. C57BL/6 control mice and *C5ar1*^−/−^ mice were co-housed and matched by age and sex. Mixed-gender groups were used. All animal studies were reviewed and approved by local authorities of the Animal Care and Use Committee (*Ministerium für Landwirtschaft, Energiewende, Umwelt und Ländliche Räume*, Kiel, Germany) according to permission number V242-7224.122-39 (36-3/15) and IACUC according to the protocol number 2013-0144 (J. Aliberti, CCHMC, Cincinnati, Ohio, USA).

### Parasites and Infection

The STAg was prepared using *T. gondii* RH tachyzoites kindly provided by A. Sher (National Institute of Allergy and Infectious Diseases (NIAID), Bethesda, USA) that were maintained on the human foreskin fibroblast cell line HS27 (ATCC CRL-1634, RRID:CVCL_0335) as previously described (32). The *T. gondii* type II strain ME49 was kindly provided by D. Schlüter (Otto von Guericke University Magdeburg, Germany). Tissue cysts were maintained through passaging in NMRI mice (Charles River) as described (33) and propagated in C57BL/6 mice 4–8 weeks before infection. C57BL/6 mice were infected by injection of 50 *T. gondii* ME49 brain cysts in 200 μl of homogenate/DPBS intraperitoneally (i.p.). The clinical course, including weight, disease severity, and survival was monitored daily.

### Disease Severity Score

Each mouse was scored daily by two people. The score was defined as follows: (1) coat, appetite, gait, posture is healthy, the mouse is alert and usually responds to external stimuli (escape behavior, reactivity); (2) the mouse is active, but moves slower than average, back slightly humped, the fur starts to become dull; (3) score two plus tilted head, ataxia or delayed righting reflex; (4) the mouse is quiet, alert and responsive, but shows a strongly hunched posture, coarse fur, reacts with movement only to external stimulation; (5) the mouse no longer responds to stimuli and is apathetic, lays on the side (34). Termination criteria were defined as a score of 5 or a loss of more than 20% of the initial body weight.

### RNA Extraction

For total RNA extraction, the whole organs were homogenized in RLT buffer (Qiagen) using an Ultra-Turrax™ homogenizer (IKA) according to the manufacturer's recommendations. RNA was extracted on spin columns using AllPrep DNA/RNA 96 Kit (Qiagen) according to the manufacturer's instructions. After extraction, RNA quantity, purity, and quality were determined by NanoDrop 1000 spectrophotometer (Thermo Scientific).

### Real-Time RT-qPCR

Reverse transcription reaction was performed after DNase I treatment of the RNA using first-strand cDNA synthesis kit (Thermo Scientific). Real-time qPCR was done using iQ SYBR Green Supermix on a CFX96 Real-Time System (Bio-Rad) using the following primers (Eurofins, Reichenwalde, Germany): β-actin 5′-GCACCACACCTTCTACAATGAG-3′ (sense) and 5′-AAATAGCACAGCCTGGATAGCAAC-3′ (antisense), IFN-γ 5′-ATGAACGCTACACACTGCATC-3′ (sense) and 5′-CCATCCTTTTGCCAGTTCCTC-3′ (antisense), IL12p35 5′-CTGTGCCTTGGTAGCATCTATG-3′ (sense) and 5′-GCAGAGTCTCGCCATTATGATTC-3′ (antisense), IL12p40 5′-CAGAAGCTAACCATCTCCTGG-3′ (sense) and 5′-AGTCCAGTCCACCTCTACAAC-3′ (antisense), IL-18 5′-TCAAAGTGCCAGTGAACCCCA-3′ (sense) and 5′-CACAGCCAGTCCTCTTACTTCA-3′ (antisense), iNOS 5′-AGCCAAGCCCTCACCTAC-3′ (sense) and 5′-AATCTCTGCCTATCCGTC-3′ (antisense) primers. The temperature profile of the quantitative (q)PCR was a follows: 95°C for 3 min, followed by 40 cycles at 95°C for 5 s, 58°C for 5 s, and 72°C for 30 s, followed by 30 cycles for 5 s with a 1°C temperature increase starting at 65°C to confirm the expected PCR products by melting curve analysis. Real-time RT-qPCR data were analyzed using CFX Manager Software 3.1 (Bio-Rad). Relative expression was calculated using the ΔΔ*Ct* method (35).

### Serum Sampling

Blood was obtained from mice through the submandibular venipuncture and collected in Microtainer tubes (BD) (36). Tubes were inverted, incubated for 30 min at room temperature for complete blood clotting, and centrifuged at 12,500 g for 5 min. Separated serum was stored at −20°C until further analysis.

### Determination of Cytokine, Chemokine, and C5a Serum Concentrations

IL-12p40 and IFN-γ were quantified by ELISA using commercial kits (R&D Systems). IL-12p70 was quantified using the V-PLEX Proinflammatory Panel 1 (mouse) kit (MSD, Rockville, USA according to manufacturer's instructions and measured using MESO Quick Plex SQ120 device (MSD). IL-1α, IL-6, IL-10, IL-23, IL-27, TNF-α, and IFN-γ were measured using the bead-based LEGENDplex™ assay (BioLegend) and analyzed on MACSQuant Analyzer 10 (Miltenyi Biotech). G-CSF, GM-CSF, CXCL1 (KC), CCL2 (MCP-1), CCL3 (MIP-1α), CCL4 (MIP-1β), and CCL5 (RANTES) were analyzed using bead-based Bio-Plex Pro Mouse Cytokine 23-plex Assay (Bio-Rad) according to manufacturer's instructions. C5a serum levels were determined as described (37). All samples were analyzed in duplicate.

### Cell Preparation From the Spleen, Mesenteric Lymph Nodes, and Peritoneum

Mice were killed under anesthesia by cervical dislocation. Isolation of cells from the spleen was performed by mechanical disruption using a 40 μm Nylon cell strainer and the plunger of a 5 ml syringe (all BD). The cell strainer was flushed three times, with 5 ml of DPBS. Cells were then incubated with RBC lysis buffer for 3 min, washed with DPBS, and used for flow cytometry analysis or DC isolation (22). CD11c^+^ cells were purified using CD11c MicroBeads, followed by MACS column separation (Miltenyi Biotech, Gladbach, Germany. Cells were cultured in complete RPMI 1640 medium (10% FBS, heat-inactivated, 100 unit/ml penicillin, 100 μg/ml streptomycin, 2 mM L-Glutamine) overnight at 37°C and 5% CO_2_. Supernatants were collected and stored at −20°C until cytokine analysis.

Mesenteric lymph nodes were removed and placed into 1.5 ml reaction tubes containing 1 ml DPBS and placed on ice. To obtain a single cell suspension, the same procedure as described for splenic cell isolation was used except that the red blood cell lysis was omitted.

Peritoneal cells were isolated as described (38). Briefly, a small incision was made in the outer skin of peritoneum, then the skin was gently pulled back to expose the inner skin lining of the peritoneal cavity. Then, 5ml of ice-cold DPBS were injected into the peritoneal cavity using a 27g needle. The peritoneum was then gently massaged to release attached cells into the peritoneal cavity. The cell suspension was carefully aspirated with the same syringe and transferred into 15 ml Falcon tube. The tube was centrifuged for 5 min at 450 g, 4°C. The supernatant was discarded and the cell pellet was used for analysis. Cells were kept on ice between manipulations.

### Brain Preparation and *T. gondii* Cyst Counting

For the brain preparation, tissue was collected and homogenized with the addition of ice-cold DPBS in a total volume of 1 ml by repeated aspiration and ejection from a 5 ml Luer-lock syringe (BD) connected to an 18, 21 g and finally a 26 g needle. The number of cysts was then counted in 10 μl of the homogenate under the coverslip using ×40 magnification.

### Flow Cytometry

Intracellular cytokine staining was performed in fixed and permeabilized cells. For cytokine accumulation, freshly isolated splenic cells were resuspended in complete RPMI medium with the addition of Brefeldin A (3 μg/ml; eBioscience) without additional stimulation. The cell suspension was incubated for 2 h (5% CO_2_, 37°C). Then, cells were labeled extracellularly, fixed and permeabilized using the Cytofix/Cytoperm kit (BD Biosciences) according to manufacturer's instruction. CD11c^+^CD8a^+^ or CD11c^+^CD11b^+^ DCs were incubated with PE-labeled anti-IL-12/23p40, whereas NK1.1^+^CD3^−^ NK cells, NK1.1^+^CD3^+^ NKT, CD3^+^CD8^+^ T cells or CD3^+^CD8^−^ T cells were incubated with PE-labeled anti-IFN-γ Abs for 30 min at 4°C, washed and analyzed by BD LSRII flow cytometer. Flow cytometry data were analyzed using FlowJo 9 software (TreeStar). Cell debris was excluded from the analysis based on the SSC-A/FSC-A signal, and doublets were excluded according to the FSC-A/FSC-H signal ratio.

### Statistical Analysis

Statistical analysis was performed using GraphPad Prism 6 (La Jolla, California, USA). Normal distribution of data was tested using the Kolmogorov-Smirnov test. Outliers were excluded from analysis using ROUT (robust regression and outlier removal) method with Q = 1%. An unpaired *t*-test was used to compare two sets of normally distributed data. Comparison between multiple groups were done by one-way or two-way ANOVA. Statistical differences regarding the survival of treatment groups were calculated using the log-rank Mantel-Cox test. Linear regression analysis with the Pearson correlation coefficient was performed to determine the relationship between two cytokines. A *p*-value < 0.05 was considered significant (^*^p < 0.05, ^**^p < 0.01, ^***^p < 0.001).

## Results

### C5aR1-Deficient Mice Are More Susceptible to Experimental *T. gondii* Infection Than wt Mice

**Figure 1:**
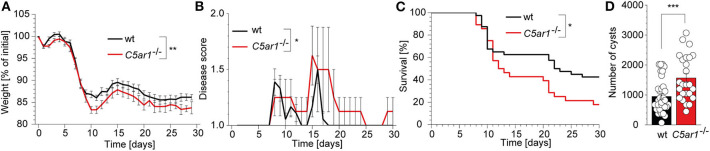
Impact of C5aR1 activation on weight loss, disease severity, survival, and parasite burden in the brain after i.p. *T. gondii* infection. Wt and *C5ar1*^−/−^ mice were infected i.p with 50 cysts of *T. gondii* type II strain ME49 and monitored for 30 days. **(A)** Relative weight change in infected wt (*n* = 30) and *C5ar1*^−/−^ mice (*n* = 23). **(B)** Change of disease score during the course of infection (wt *n* = 18, *C5ar1*^−/−^ *n* = 9). **(C)** Survival of wt and *C5ar1*^−/−^ mice post-infection. Statistical differences in survival between wt (*n* = 40) and *C5ar1*^−/−^ (*n* = 28) mice were calculated using the Log-rank Mantel-Cox test. **(D)** Cyst count in brain tissues 30 days after infection in surviving wt and *C5ar1*^−/−^ mice as counted in the brain homogenate using light microscopy (wt *n* = 35, *C5ar1*^−/−^ *n* = 25). Values shown in **(A,B,D)** are the mean ± SEM; differences between groups were compared by unpaired *t*-test, *p < 0.05, **p < 0.01, ***p < 0.001.

The gut microbiota actively contributes to the innate immune sensing during oral *T. gondii* infection through the activation of several TLRs including TLR2, TLR4, and TLR9 at the site of invasion (39). Thus, we decided to use an intraperitoneal infection model that primarily depends on the direct sensing of parasite-derived antigens by the intracellular TLR11/12 dimers (40). We infected wt and *C5ar1*^−/−^ mice with 50 brain cysts of the type II *T. gondii* strain ME49 i.p. and recorded the weight (Figure 1A), clinical score (Figure 1B), and survival time (Figure 1C) of these animals for 30 days. At the end of the experiment, we sacrificed the surviving mice and determined the parasite load by direct microscopic counting of cysts in the brain homogenate (Figure 1D). *C5ar1*^−/−^ mice suffered from a significantly higher relative weight loss, disease severity during infection (Figures 1A,B) that was associated with a ~20% increase in the mortality rate in the *C5ar1*^−/−^ group as compared to control mice (Figure 1C). Further, the number of parasite cysts in the brain of those *C5ar1*^−/−^ animals that survived the *T. gondii* infection for 30 days was significantly higher than that of wt mice (Figure 1D). Together, these data suggest that parasite-sensing by the complement system and consecutive C5a generation is critical for the development of appropriate innate immune responses during the first 30 days after *T. gondii* infection.

### C5a Serum Concentrations Increase During the First 5 Days After *T. gondii* Infection

**Figure 2:**
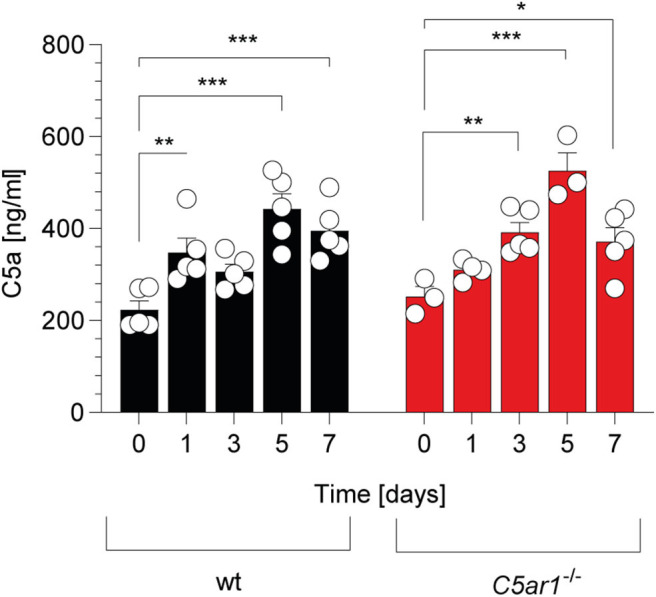
Kinetic of C5a serum concentrations in wt and *C5ar1*^−/−^ mice during the first 7 days post-i.p. *T. gondii* infection. Serum samples were obtained before and 1, 3, 5, and 7 days after infection. Cytokine levels were determined by ELISA. Values shown are the mean ± SEM, *n* = 3–5. Differences between control and infected groups were compared by ANOVA with Sidak's *post-hoc* test, *p < 0.05, **p < 0.01, ***p < 0.001.

In support of this view, previous studies have demonstrated that C5 is an indispensable part of complement-mediated *T. gondii* elimination (14, 15). C5 can be cleaved into the AT C5a and C5b by canonical and non-canonical pathways (41). We determined the kinetics of C5a generation in the serum of wt and *C5ar1*^−/−^ mice during the acute phase of infection. Before the infection, C5a serum levels were in the range of 200-250 ng/ml (Figure 2) in wt and *C5ar1*^−/−^ mice. During the first week after infection, C5a concentrations increased similarly in both mouse strains, peaked at day 5, and started to decline on day 7 (Figure 2), demonstrating significant early systemic complement activation after i.p. *T. gondii* infection.

### *T. gondii* Infection Is Associated With a Lower Frequency of Neutrophils in the Peritoneal Cavity of C5aR1-Deficient Mice

**Figure 3:**
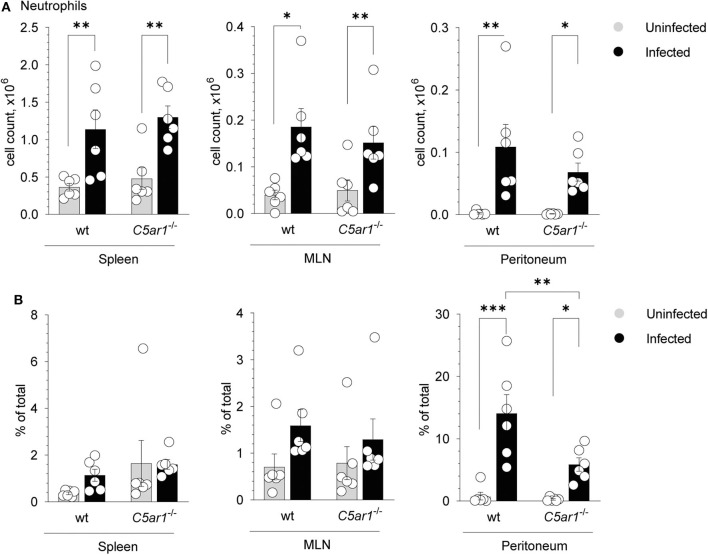
Impact of C5aR1 activation on neutrophil recruitment into the spleen, mesenteric lymph nodes and the peritoneum. **(A,B)** Number **(A)** or frequency **(B)** of neutrophils in the spleen (left panel), mesenteric lymph nodes (MNL; middle panel) and peritoneum (right panel) of uninfected and *T. gondii*-infected wt and *C5ar1*^−/−^ mice. Values shown are the mean ± SEM; *n* = 6/group. Differences between groups were determined by ANOVA and Sidak's *post-hoc* test, *p < 0.05, **p < 0.01, ***p < 0.001.

Given that C5aR1-deficient mice suffered from more severe clinical symptoms, increased mortality, and higher parasite burden when compared to wt mice, we aimed to delineate the mechanisms of higher parasite susceptibility. Previously, it has been shown that neutrophils play an important role in *T. gondii* infection (9, 42). Given that neutrophils express C5aR1 at high levels and migrate strongly toward C5a (23), we determined neutrophil numbers and frequencies in the spleen, mesenteric lymph nodes (MLN), and the peritoneum in uninfected mice and 5 days after *T. gondii* infection. Neutrophils were identified as Lin^−^Ly6G^+^ in the different organs (Supplementary Figure 1). As shown in Figure 3A, neutrophil numbers significantly increased in the spleen, MLN, and peritoneum in wt and *C5ar1*^−/−^ mice after infection Importantly, while neutrophil numbers were similar in the spleen and MLN of wt and *C5ar1*^−/−^ mice, we found slightly lower cell numbers in the peritoneum of *C5ar1*^−/−^ mice when compared to wt animals. More strikingly, the frequency of neutrophils was markedly reduced in C5aR1-deficient animals (Figure 3B) suggesting that C5aR1 activation contributes to the recruitment of neutrophils into the peritoneum in response to *T. gondii* infection. TLR11-independent production of IFN-γ from neutrophils has been described in response to *T. gondii* (42). However, we observed no IFN-γ production in neutrophils from spleens of wt and *C5ar1*^−/−^ mice 5 days after infection (data not shown).

### Low IL-12 Family Cytokine and Chemokine Serum Levels in C5aR1-Deficient Mice Are Associated With Low IFN-γ Serum Concentrations 7 Days After *T. gondii* Infection

**Figure 4:**
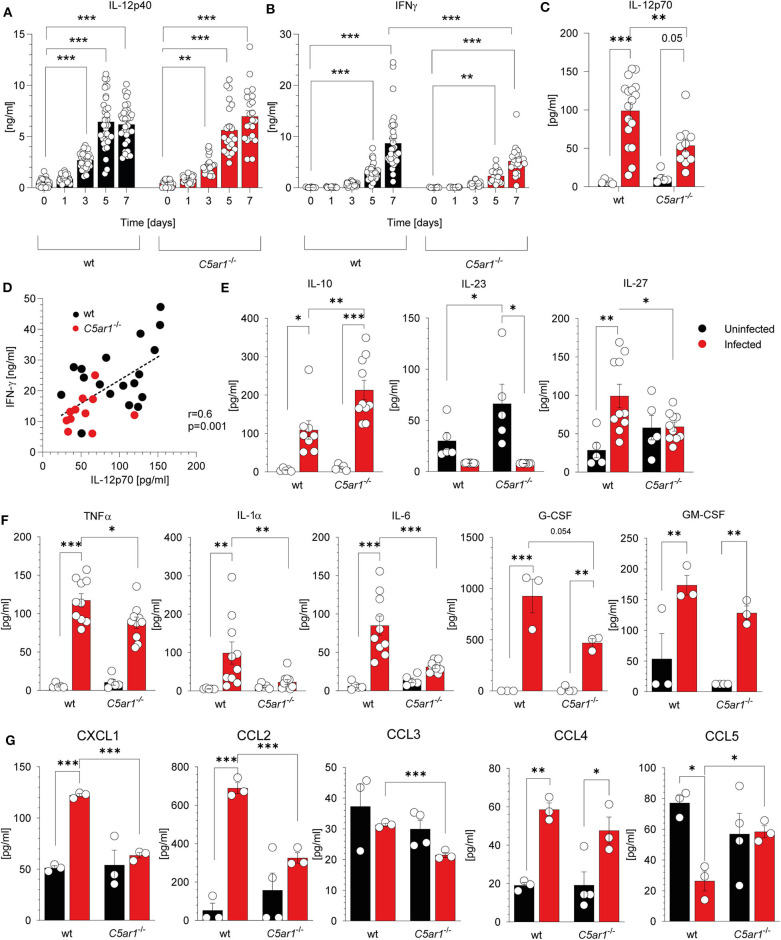
C5aR1 activation drives systemic cytokine and chemokine production during the early phase of *T. gondii* infection. **(A)** Kinetics of IL-12p40 and **(B)** IFN-γ serum concentrations in wt and *C5ar1*^−/−^ mice after *T. gondii* infection. Serum samples were obtained before and 1, 3, 5, and 7 days after infection. Cytokine levels were determined by ELISA. The data are from three independent experiments, *n* = 22–33/condition. **(C)** IL-12p70 cytokine serum concentrations in wt and *C5ar1*^−/−^ mice 7 days after *T. gondii* infection as determined by bioplex assay (Meso Scale Discovery); *n* = 10–17/condition. **(D)** Linear regression analysis between IL-12p70 and IFN-γ serum concentrations 7 days after infection. The *p*-value was calculated using the Pearson's correlation coefficient, *n* = 29 pairs. **(E)** IL-10, IL-23, and IL-27 cytokine serum concentrations in wt and *C5ar1*^−/−^ mice 7 days after *T. gondii* infection; *n* = 10–17/condition. **(F)** Serum concentrations of TNF-α, IL-1α, IL-6, G-CSF, and GM-CSF determined in wt and *C5ar1*^−/−^ mice 7 days after infection. Cytokine concentrations were determined by LEGENDplex™ assay (BioLegend), *n* = 10/group. **(G)** Serum concentrations of CXCL1 (KC), CCL2 (MCP-1), CCL3 (MIP-1α), CCL4 (MIP-1β), CCL5 (RANTES) in wt and *C5ar1*^−/−^ mice 7 days after infection. The cytokines and chemokines were determined by Bio-Plex assay (Bio-Rad), *n* = 3/group. Values shown in **(A–C)** and **(E–G)** are the mean ± SEM, Differences between groups were determined by ANOVA and Sidak's *post-hoc* test. *p < 0.05, **p < 0.01, ***p < 0.001.

Previously, we demonstrated that C5aR1 controls the production of cytokines and chemokines that are associated with resistance to *T. gondii* infection (43–46). Here, we focused on the impact of C5aR1 activation on IL-12 as the main driver of IFN-γ production from NK cells and the development of protective Th1 immunity (4, 10, 47). IL-12 is a heterodimer comprising the p40 and p35 subunits. First, we determined the kinetics of systemic IL-12p40, and IFN-γ concentrations in wt and *C5ar1*^−/−^ mice during the first 7 days after *T. gondii* infection. The IL-12p40 concentration steadily increased after infection and reached a maximum of 6.2 ± 1.96 (wt) or 6.98 ± 2.67 ng/ml (*C5ar1*^−/−^) 5 days after infection in both mouse strains (Figure 4A). IFN-γ levels started to increase on day three and reached a maximum on day 7. Strikingly, the IFN-γ concentrations in wt mice were significantly higher than in *C5ar1*^−/−^ mice on day 7 (8.68 ± 5.41 vs. 5.22 ± 2.73 ng/ml) (Figure 4B). Next, we determined IL-12p70 serum concentrations in wt and *C5ar1*^−/−^ mice at day seven after *T. gondii*. We observed significantly higher levels of IL-12p70 in wt than in *C5ar1*^−/−^ animals (Figure 4C), sugessting that C5a/C5aR1 axis activation controls early IFN-γ production at the level of IL-12 production. The significant correlation between IL-12 and IFN-γ serum concentrations further corroborates this view (Figure 4D). In addition to IL-12p70, we also determined the impact of C5aR1 activation on the production of other IL-12 family cytokines, including IL-23 and IL-27. While IL-12p70 is critical for early IFN-γ production by NK cells after primary infection, IL-23 has recently been found to contribute to protective immunity against secondary *T. gondii* infection (48). IL-23 serum levels in *T. gondii*-infected mice were minor and much lower than in naïve wt or *C5ar1*^−/−^ mice, suggesting either reduced production or high consumption (Figure 4E). Interestingly, we found a significant increase in IL-27 serum concentrations in wt but not in *C5ar1*^−/−^ mice after infection (Figure 4E). IL-27 is known to regulate T cell responses and has previously been shown to reduce the immunopathology in experimental murine toxoplasmosis (49). We also measured TNFα (50), IL-1α (51), IL-6 (52), and IL-10 (10, 53) as these cytokines have also been demonstrated to confer protection from acute *T. gondii* infection (Figures 4E,F). In particular, TNF-α is essential during early *T. gondii* infection in mice, as it is also required for NK cell priming (50). Similar to the IL-12 family cytokines, we detected significantly lower amounts of TNF-α, IL-1α, and IL-6 in the serum of C5aR1-deficient animals in comparison to the wt group (Figure 4F). Further, G-CSF serum levels were lower in *C5ar1*^−/−^ mice, whereas GM-CSF levels were similar (Figure 4F). In contrast, we observed significantly higher serum levels of IL-10 in C5aR1-deficient mice (Figure 4E). Of note, IL-10 induced by *T. gondii* was found to suppress acute pathological inflammation that causes tissue necrosis and early mortality (53–55). Additionally, chemokine induction was low in *C5ar1*^−/−^ mice as reflected by lower serum levels of CXCL1 (KC), CCL2 (MCP-1), and CCL3 (MIP-1α) when compared to those of wt mice whereas and CCL4 (MIP-1β) levels were similar between wt and *C5ar1*^−/−^ mice (Figure 4G). Interestingly, serum levels of CCL5 (RANTES) were elevated in *C5ar1*^−/−^ animals in comparison to wt animals (Figure 4G).

### Activation of C5aR1 Axis in the Spleen and the Brain Is Required for the Expression of IFN-γ and iNOS

**Figure 5:**
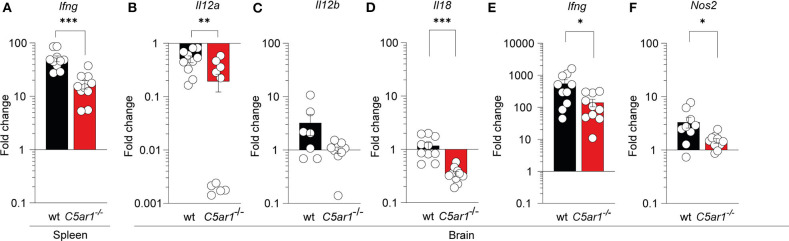
Impact of C5aR1 deficiency on *Ifng mRNA expression in the spleen and brain and on Il12a, Il12b, Il18*, and *Nos2* mRNA expression in the brain during the early phase of *T. gondii* infection. *Ifng* mRNA transcription in the spleen **(A)**, and *Il12a* **(B)**, *Il12b* **(C)**, *Il18* **(D)**, *Ifng* **(E)**, and *Nos2* **(F)** in the brain of wt and *C5ar1*^−/−^ mice 7 days after *T. gondii* infection as determined by RT-qPCR. The data show the relative change in mRNA transcripts in comparison to uninfected mice; the reference value for uninfected mice was set as 1, *n* = 10/group. Values shown are the mean ± SEM, Differences between groups were determined by unpaired *t*-test, *p < 0.05, **p < 0.01, ***p < 0.001.

It has been previously demonstrated that IL-12 and IFN-γ are essential in controlling *T. gondii* tachyzoite invasion (4) whereas inflammasome activation and IL-18 production can enhance IL-12-driven IFN-γ production (11, 56, 57). Further, nitric oxide (NO) production form IFN-g-activated macrophages in response to iNOS is a crucial effector molecule of antimicrobial activty (12). Thus, we determined whether local C5aR1 activation affects the transcription of IFN-γ (*Ifng*) in the spleen and brain as well as IL-12p35 (*Il12a*), IL-12p40 (*Il12b*), IL-18 (*Il18*), and iNOS (*Nos2*) in the brain of *T. gondii*-infected animals on day seven after infection using RT-qPCR (Figures 5A–F). Baseline cytokine gene and *Nos2* expression in wt and *C5ar1*^−/−^ mice were similar except *Il18*, which was somewhat reduced in *C5ar1*^−/−^ mice (Supplementary Figure 2). We observed a strong upregulation of *Ifng* expression upon infection, which was >50-fold in the spleen (Figure 5A) and >500-fold in the brain (Figure 5E) of wt mice. Consistent with our observations in the serum, the *Ifng* gene expression in the spleen and brain of *C5ar1*^−/−^ mice was significantly lower than that in wt mice (Figures 5A,E). The markedly increased *Ifng* expression in the brain was associated with a 3-fold increase in *Il12b*, whereas *Il12a* expression was decreased, and that of *Il18* was unchanged in wt mice (Figures 5C–F). *Il12a, Il12b*, and *Il18* expression in the brain of *C5ar1*^−/−^ mice was consistently lower than that in wt mice (Figures 5C–E). Finally, we determined *Nos2* mRNA expression in the brain, which encodes the iNOS effector molecule that is crucial for parasite control during the chronic stage of infection (13). Importantly, we found a 3-fold upregulation of *Nos2* mRNA expression in the brain of infected wt when compared to uninfected control mice, whereas *C5ar1*^−/−^ mice poorly induced *Nos2* transcripts in the brain (Figure 5F).

### C5aR1 Activation Has a Moderate Impact on the Frequency and Number of IFN-γ-Producing NK and NKT Cells in the Spleen

**Figure 6:**
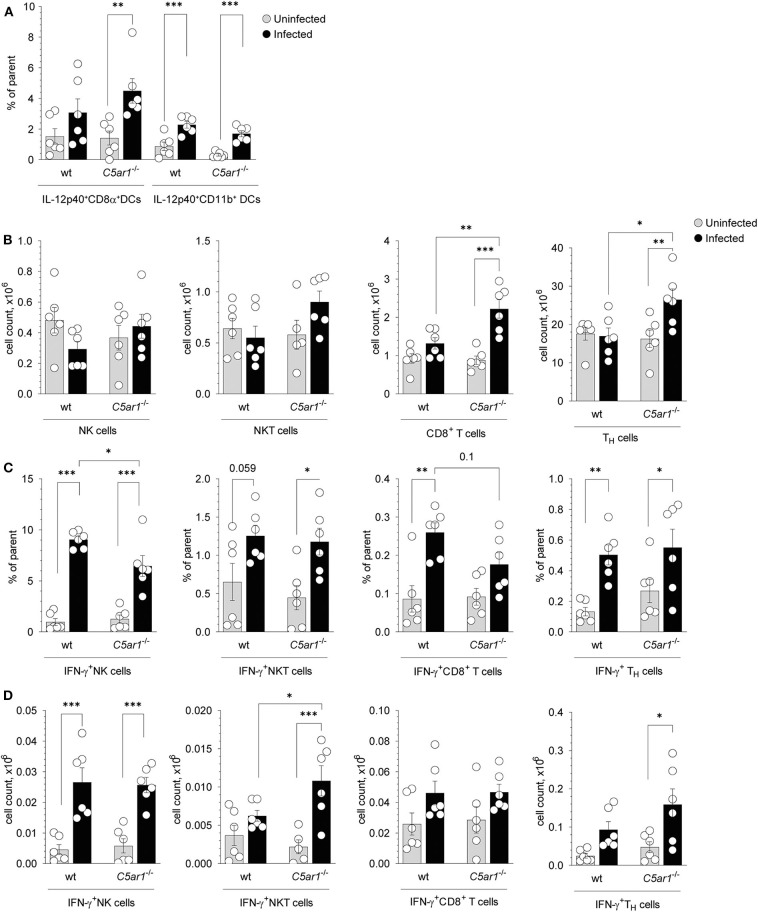
Impact of C5aR1 activation on IL-12p40 production from splenic DCs and IFN-γ production from splenic NK, NKT, and T cells during acute *T. gondii* infection. **(A)** Frequencies of IL-12p40^+^ CD8α^+^ (left) and IL-12p40^+^ CD11b^+^ (right) DCs in the spleen of uninfected and *T. gondii*-infected wt and *C5ar1*^−/−^ mice, *n* = 6/group. **(B–D)** Total cell numbers **(B)**, frequencies **(C)**, and numbers **(D)** of IFN-γ^+^ NK (left), NKT (middle-left), CD8^+^ T cells (middle-right), and CD8^−^ T_H_ cells (right) in the spleen of uninfected and *T. gondii* infected wt and *C5ar1*^−/−^ mice, *n* = 6/group. Cytokine production was assessed at day 5 after *T. gondii* infection by the intracellular staining. Cells were incubated *ex vivo* for 2 h in the presence of 3 μg/ml of Brefeldin A without additional stimulation. Values shown are the mean ± SEM, Differences between groups were determined by ANOVA and Sidak's *post-hoc* test, *p < 0.05, **p < 0.01, ***p < 0.001.

CD8α^+^ DCs in the spleen are critical for parasite sensing and the main source of IL-12 production during *T. gondii* infection (6, 40). Previously, we demonstrated that C5aR1 is expressed on CD8α^+^ but not on CD11b^+^ conventional DCs in the mouse spleen using a GFP-C5aR1 knock-in mouse (23). Here we used intracellular cytokine staining to determine the frequencies of IL-12p40-producing CD8α^+^ and CD11b^+^ DCs in the spleen 5 days after *T. gondii* infection (Supplementary Figure 3). We found upregulated IL-12p40-expression in CD8^+^ DCs and CD11b^+^ DCs from wt and *C5ar1*^−/−^ mice. However, the frequencies of IL-12p40 expressing DCs were similar in wt and *C5ar1*^−/−^ animals (Figure 6A). Next, we assessed the total numbers of IFN-γ producing NK, NKT, as well as CD8^+^ and CD8^−^ T cells in the spleens of the same animals (Supplementary Figure 4, Figures 6B–D). We found no differences in the total numbers of NK and NKT cells in wt and *C5ar1*^−/−^ mice before or after *T. gondii* infection. In contrast, the number of CD8^+^ T cells and CD8^−^ T_H_ cells increased in *C5ar1*^−/−^ but not in wt mice (Figure 6B). Within the group of NK cells, we observed a markedly increased frequency of IFN-γ^+^ NK cells from 0.97 ± 0.83% or 1.26 ± 0.98% in uninfected wt and *C5ar1*^−/−^ mice to 9.05 ± 0.82% or 6.46 ± 2.49% in infected wt or *C5ar1*^−/−^ mice (Figure 6C). The frequency of IFN-γ^+^ NKT cells increased modestly in response to infection in wt and C5ar1^−/−^ mice (Figure 6C). Also, we found a moderate increase in the frequency of IFN-γ^+^ CD8^−^ or CD8^+^ T cells in wt and *C5ar1*^−/−^ mice (Figure 6C). Of note, the frequency of IFN-γ^+^ CD8^+^ T cells was somewhat higher in wt than in *C5ar1*^−/−^ mice. Importantly, the frequency of IFN-γ^+^ NK cells was significantly lower in *C5ar1*^−/−^ mice as compared with wt mice, whereas the frequencies of IFN-γ-expressing NKT and CD8^−^ T_H_ cells were similar in wt and *C5ar1*^−/−^ mice (Figure 6C). Interestingly, we did not detect C5aR1 expression in naïve spleen-derived NK cells using the GFP-C5aR1 knock-in mice (23), suggesting that the effect of C5a on IFN-γ production from NK is indirect and likely mediated by IL-12-producing DCs residing in the spleen.

In addition to the frequencies, we also determined the numbers of IFN-γ^+^ NK, NKT, and T cells in the spleen (Figure 6D). Similar to the frequency, we found a strong increase in the number of IFN-γ^+^NK cells after infection, both in wt and in *C5ar1*^−/−^ mice (Figure 6D). However, in contrast to the frequency, the numbers of NK cells were similar in wt and *C5ar1*^−/−^ mice. The increase in NKT cell numbers was only minor in wt mice but massive in *C5ar1*^−/−^ animals (Figure 6D) and thus significantly higher than in wt mice. However, as compared to IFN-γ^+^ NK or -T cells, the number of IFN-γ^+^ NKT cells was much lower. The number of IFN-γ^+^ CD8^−^ T_H_ and CD8^+^ T cells increased moderately, and we observed no differences between wt and *C5ar1*^−/−^ mice despite the upregulation in the total cell numbers of these populations in *C5ar1*^−/−^ mice (Figures 6B,D). The number of IFN-γ^+^ CD8^−^ T cells after infection in wt or *C5ar1*^−/−^ mice was 5- to 10-fold higher than that of IFN-γ^+^ NK or NKT cells and 2- to 3-fold higher than that of IFN-γ^+^ CD8^+^ T cells (Figures 6B,D). Thus, C5aR1 activation impacts mainly on the frequency but not the total number of IFN-γ^+^ NK cells. Also, it affects the number and frequency of NKT cells but has no impact on IFN-γ^+^ T cell numbers and frequencies.

### C5aR1 Activation Is Critical for IL-12 Production From Spleen-Residing DCs in Response to *T. gondii* Antigen Challenge

**Figure 7:**
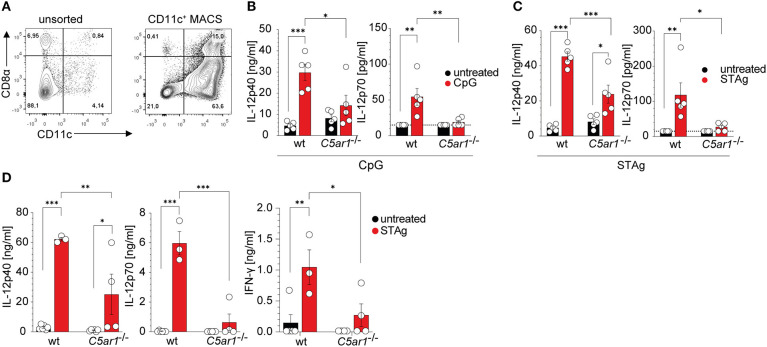
The C5a/C5aR1 axis controls TLR9 and STAg-induced IL-12 production from spleen-residing DCs. **(A)** Dot plots showing the frequencies of unsorted (left) and CD11c^+^ MACS positively selected (right) splenic DCs. The numbers in the four quadrants show the frequencies of the individual cell populations. **(B)** Impact of CpG ODN1668 (1 μg/ml) or **(C)** STAg (1 μg/ml) stimulation of splenic DCs (1 × 10^6^) from wt or *C5ar1*^−/−^ mice on IL-12p40 (left) and IL-12p70 (right) production. The dotted line depicts the detection limit of the assay, *n* = 5/group. **(D)** Impact of STAg injection (i. p., 100 μg, 6 h) on IL-12p40 (left), IL-12p70 (middle), and IFN-γ (right) serum concentrations in wt and *C5ar1*^−/−^ mice. The dotted line depicts the detection limit of the assay, *n* = 3–6/group. Values shown in **(B–D)** are the mean ± SEM, differences between groups were determined by ANOVA and Sidak's *post-hoc* test, *p < 0.05, **p < 0.01, ***p < 0.001.

Given that C5aR1 is expressed on DCs and that DCs are essential producers of IL-12, we assessed the potential of C5aR1 activation to drive IL-12 production in spleen-residing DCs. We used CD11c^+^ MACS-enriched DCs, which comprised ~10% CD8α^+^ DCs and in total ~90% of CD11c^+^ cells in wt and *C5ar1*^−/−^ mice as determined by flow cytometry (Figure 7A, Supplementary Figure 3B). DCs from naïve wt or *C5ar1*^−/−^ mice were stimulated with either the TLR9 ligand CpG ODN1668 or STAg, both of which signal through MyD88-dependent pathways to induce their effector functions. As expected, CpG ODN1668 stimulation resulted in significant production of IL-12p40 and IL-12p70. In contrast, we found only a minor increase in IL-12p40 and IL-12p70 production in the supernatants from *C5ar1*^−/−^ sDCs (Figure 7B). IL-12p40 as well as IL-12p70 production in wt DCs was significantly higher than in *C5ar1*^−/−^ DCs (Figure 7B). Similar to CPG, STAg stimulation induced strong IL-12p40 and IL-12p70 production in spleen DCs (Figure 7C). In *C5ar1*^−/−^ DCs, the STAg challenge induced increased IL-12p40 production. However, this increase did not translate into enhanced production of IL-12p70. IL-12p40 and IL-12p70 production of IL-12p40 and IL12-p70 were significantly higher in wt than in *C5ar1*^−/−^ DCs (Figure 7C).

To assess *ex vivo* cytokine production, we injected naïve wt and *C5ar1*^−/−^ mice i.p. with 100 μg of STAg. After 6 h, we took blood samples and determined IL-12p40, IL-12p70, and IFN-γ serum concentrations. Similar to what we had observed in the case of TLR9 stimulation, we found high concentrations of systemic IL-12 and IFN-γ in the serum of wt mice (Figure 7D). In contrast, *C5ar1*^−/−^ mice failed to upregulate serum levels of IL-12p40, IL-12p70, and subsequently, IFN-γ (Figure 7D), which is crucial for the induction of effector mechanisms against *T. gondii* infection (9).

## Discussion

The biologic relevance of complement activation in *T. gondii* infection is ill-defined. The extracellular phase within the life cycle of *T. gondii* suggests that the parasite may have evolved mechanisms to evade complement-mediated sensing and attack by the MAC of the terminal pathway. Recent findings uncovered that *T. gondii* type I and II strains not only activate complement by the AP and LP but bind C4BP and FH to limit complement activation at the parasite surface to survive complement attack (16). In line with this observation, we found activation of the complement cascade, which resulted in the generation of C5a in the circulation of wt and *C5ar1*^−/−^ animals peaking on day five after infection. On the other hand, the parasite seems to exploit complement activation as an essential pathway to limit the uncontrolled proliferation of tachyzoites and the killing of the host. After infection with 40 cysts of the *T. gondii* type II strain ME49 > 75% of C3-deficient mice succumbed to infection whereas all wt mice survived the observation period of 30 days suggesting that pathways downstream of C3 tip the balance (16) between fatal acute and persistent infection which guarantees the transmission of the parasite to new hosts. Also, mice lacking C5aR1 and C3aR died 12 days after i.p. infection with 20 cysts of *T. gondii*, whereas all wt mice survived >50 days (26). The individual contribution of C3aR or C5aR1 to this effect remained unclear. We found a significantly higher disease severity score and higher weight loss in C5aR1-deficient animals, which was associated with a higher mortality rate when compared to wt controls. In 20% of *C5ar1*^−/−^ mice that survived the 30 days' observation period, the parasite burden in the brain was significantly higher than that observed in the 40% of the surviving wt mice, suggesting that C5aR1 activation is an essential immune mechanism to control the parasite burden in the brain.

In the search for mechanisms underlying the impaired resistance of *C5ar1*^−/−^ mice to *T. gondii* infection, we monitored IL-12p40, and IFN-γ serum levels during the first 7 days after infection. While the initial increase in IFN-γ production was similar between wt and *C5ar1*^−/−^ mice, we found significantly lower IFN-γ serum concentrations 7 days after infection. Surprisingly, the difference in IFN-γ production was not matched by the IL-12p40 serum concentrations. However, when we determined IL-12p70 on day 7 after infection, we found a significantly lower cytokine production in *C5ar1*^−/−^ as compared with wt mice. These data suggested that the C5a/C5aR1 axis controls early IFN-γ production at the level of IL-12p35 production. However, when we determined *Il12a* and *Il12b* gene expression in the brain, we not only observed lower *Il12a* but also *Il12b* expression in *C5ar1*^−/−^ mice. Also, STAg-induced IL-12p40 and IL12p70 production was significantly lower in *C5ar1*^−/−^ than in wt DCs and in *C5ar1*^−/−^ mice after *in vivo* administration of STAg suggesting that C5aR1 activation controls both IL-12p35 and IL-12p40 production in *T. gondii* infection mainly through its impact on DCs. Since IL-12p40 serum levels were not affected by C5aR1 deficiency, other pathways seem to add to IL-12p40 serum production. Previously, a similar regulatory effect of C5a on the IL-12/IFN-γ axis has been shown in models of sepsis and chronic inflammatory diseases (58). When we assessed the induction of the IL-12 family cytokines IL-23 and IL-27, we detected only minor IL-23 serum concentrations in wt and *C5ar1*^−/−^ mice and no IL-17 production (data not shown). The available data suggest that IL-12/23p40 and IL-12p35, but not IL-23p19, drive IFN-γ production during early primary *T. gondii* infection (59) and that IL-23 contributes to IFN-γ-dependent protection during a secondary *T. gondii* infection (48). Noteworthy, IL-12p40 homodimer formation has been described to inhibit IL-12p70-mediated IFN-γ production in a dose-dependent manner (60). The role of IL-12p40 homodimers in *T. gondii* infection has not been studied yet. In contrast to IL-23, IL-27 serum levels sharply increased after infection in wt and to a much lower extent in *C5ar1*^−/−^ animals. IL-27Rα (WSX-1) signaling controls T cell proliferation and immune cell infiltration in *T. gondii* infection (61). The reduced IL-27 serum levels in *C5ar1*^−/−^ animals during the acute stage of infection might explain the increased numbers of CD8^+^ and CD8^−^T_H_ cells in the spleen of such mice as compared with wt animals. However, however it did not translate into higher frequencies or numbers of IFN-γ^+^ T cells (as shown in Figures 6B,C).

While IL-12 is necessary and sufficient to mount an appropriate IFN-γ response to *T. gondii* infection, IL-18 can synergize and enhance IL-12-mediated resistance to the parasite (11, 56, 57). Further, in the absence of TLR11, caspase-1 and-11 drive robust IFN-γ production from Th1 cells which is critical for host survival in response to *T. gondii* infection (62). We found no increase in local *Il18* gene expression in the brain of *T. gondii*-infected wt mice and markedly reduced Il18 expression in *C5ar1*^−/−^ animals, pointing toward a regulatory impact of C5aR1 activation on local IL-18 production in the brain. Of note, local *Il18* gene expression in *C5ar1*^−/−^ mice was already somewhat reduced in non-infected mice indicating regulation of steady state *Il18* gene expression.

In addition to the IL-12 family cytokines, the absence of C5aR1 signaling impaired IL-1α, IL-6, and TNF-α production in acutely infected mice. TNF-α and IL-1α are critical for survival during acute murine toxoplasmosis (51). IL-6 has been shown to promote IL-17 production from NK cells during *T. gondii* infection (52). However, as already pointed out, we did not detect IL-17 in serum of infected mice (data not shown). In addition to IL-1α, IL-6 and TNF-α, systemic levels of CXCL1 (KC), CCL2 (MCP-1), and CCL3 (MIP-1α) were significantly lower in *C5ar1*^−/−^ than in wt mice. CXCL1 is a strong chemotaxin for neutrophils via CXCR2. It is produced in large amounts by peritoneal mesothelial cells in response to IL-1β and TNF-α (63). In line with the reduced TNF-α and CXCL1 serum levels, we found reduced recruitment of neutrophils into the peritoneum of C5aR1-deficient mice, whereas we observed no differences in macrophage numbers in the peritoneum, spleen, or MLNs in *C5ar1*^−/−^ mice (data not shown). Interestingly, CCL5 (RANTES) serum levels were higher in *C5ar1*^−/−^ mice than in wt animals. CCR5, the cognate receptor for RANTES, can form heterodimers together with C5aR1 (64), which contributes to CCR5-mediated HIV entry into macrophages (65). However, the functional relevance of heterodimer formation in *T. gondii* infection remains to be determined, in particular, as CCR5 has also been described as a ligand for *T. gondii*-derived cyclophilin 18 (3). Of note, cyclophilin 18-mediated activation of CCR5 on CD8α^+^ DCs in the spleen can also drive IL-12 production (3).

To determine the source of serum IFN-γ, we performed a transcriptional analysis of this cytokine in the spleen. We found a massive upregulation of *lfng* mRNA in wt mice that was significantly impaired in *C5ar1*^−/−^ animals suggesting that C5aR1 activation of spleen cells regulates the production of IFN-γ. Also, we assessed the mRNA expression of *Ifng* and the IFN-γ-inducing *Il12a* and *Il12b* as well as *Il18* genes in the brain. Similar to the spleen, *Ifng* gene expression was markedly increased in wt mice and to a lesser extent in wt animals. This was associated with decreased *Il12a* and *IL12b* gene expression in C5aR1-deficient mice as compared with wt animals. Also, we detected a significant downregulation of *Il18* gene transcription in the brain of C*5ar1*^−/−^ but not of wt mice. However, we already noted a lower number of *Il18* gene transcripts in naïve *C5ar1*^−/−^ mice. Importantly, we found upregulation of *Nos2* mRNA expression in the brain of wt but not *C5ar1*^−/−^ mice during early infection in comparison to the naïve state. The reduced *Nos2* production will likely result in the lower generation of nitrogen intermediates required for parasite elimination by microglia/macrophages during bradyzoite conversion. It may cause the increased parasite burden in the brain that we have observed in *C5ar1*^−/−^ mice on day 30 (13).

To identify the cellular sources of IL-12 and IFN-γ during the early phase of *T. gondii* infection, we determined intracellular IL-12p40 production in CD8α^+^ and CD11b^+^ DCs and IFN-γ production in NK, NKT, and T cells from wt and *C5ar1*^−/−^ mice. CD8α^+^ DCs in the spleen is the main source of IL-12 (3). Previously, we identified C5aR1, but not C3aR or C5aR2 expression on CD8α^+^ DCs in the spleen using transgenic AT receptor reporter mice; none of these receptors was expressed on naïve or activated T cells (18, 22, 23, 30). The frequencies of IL-12p40^+^ CD8α^+^ and CD11b^+^ DCs increased to a similar extent after infection in wt and *C5ar1*^−/−^ mice. Interestingly, C5a downregulates the expression of IRF1 and IRF8 (ICSBP) in a *Leishmania major* model (44). IRF8 is a transcription factor essential for the development of CD8α^+^ DCs (66), and together with NF-κB specifically mediates profilin-induced IL-12 secretion from DCs (40, 67), providing a potential link for TLR11/12 and C5aR1 cross-talk that could be explored in future studies.

Regarding the IFN-γ production, we identified 8–10% of wt NK cells as IFN-γ producers, whereas around 6% of NK cells from C5aR1-deficient mice were IFN-γ^+^ during acute *T. gondii* infection. IFN-γ production from NK cells during acute *T. gondii* infection is critical to mount an appropriate Th1 response (10). Animals in which NK cells are depleted succumb to *T. gondii* infection (47). In line with the reduced IL-12p70 serum levels and the reduced IL-12p70 production from splenic DCs after STAg activation, these findings suggest that C5aR1 activation contributes to early activation of NK cells in response to *T gondii* infection through a paracrine mechanism, i.e., IL-12 production in spleen-residing DC, similar to the regulation of T cell differentiation (29). In support of this view, we found no detectable C5aR1 expression on murine NK cells taking advantage of GFP-C5aR1 knock-in mice (22, 30, 68). This is different from what we have recently found for C5aR2, which is expressed in a subset of splenic and blood NK cells (22). Specific activation of C5aR2 on NK cells suppressed IL-12/IL-18-induced IFN-γ production (22).

In addition to NK cells, we observed that already 0.5% of wt and *C5ar1*^−/−^ CD8^−^ T_H_ cells were IFN-γ producers. Although the frequency of IFN-γ-producing CD8^−^ T_H_ cells was 16- to 20-fold lower than that of IFN-γ-producing NK cells, the overall number of IFN-γ-producing T cell was still 3- to 5-fold higher than that of NK cells given the much higher number of T cells in the spleen. Also, the frequency of IFN-γ^+^ CD8^+^ T cells from wt and *C5ar1*^−/−^ mice was much lower (~50-fold) than that of IFN-γ^+^ NK cells, whereas the total number of IFN-γ^+^ CD8^+^ T cells from wt and *C5ar1*^−/−^ mice was comparable to IFN-γ^+^ NK cells. Thus, C5aR1 activation does not seem to regulate IFN-γ production from T cells in the spleen. Similarly, C5aR1 activation does not seem to affect the frequency of IFN-γ^+^ NKT cells. IFN-γ production by NKT cells plays an important role in the initiation of the inflammatory bowel response after oral *T. gondii* infection (69). However, the contribution of NKT cells to IFN-γ production after peritoneal *T. gondii* infection is unclear. Our data suggest that NKT cells become activated after i.p. *T. gondii* infection and add to the early IFN-γ production after acute infection.

Multiple studies showed a robust cell-specific impact of C5a/C5aR1 axis activation on the regulation of IL-12 production in different disease models (44, 70–73). C5a stimulation induces IL-12 production from activated monocytes and macrophages (71, 73, 74). However, C5a suppresses TLR-induced IL-12 production from macrophages in a dose- and time-dependent manner (44, 71, 72). Paradoxically, complete ablation of C5aR1 in genetically modified mice does not revert this effect but reduces the amount of secreted IL-12 (45, 68, 70, 71). In line with these data, *C5ar1*^−/−^ DCs showed markedly reduced TLR-induced IL-12 production in response to CpG and STAg stimulation *in vitro*. This effect was also present upon STAg injection *in vivo* and was associated with a decreased IFN-γ production, which is crucial for triggering effector mechanisms that combat intracellular parasite infection (9, 75).

Collectively, we uncovered a critical protective role for C5a/C5aR1 axis activation during the early phase of *T. gondii* infection. We propose a model, in which early C5a generation activates the C5aR1/C5a axis on spleen-residing CD8α^+^ DCs to synergize with *T. gondii* antigen-driven IL-12 production. This IL-12 production is critical to activate NK cells for IFN-γ production eventually activating phagocytic cells for iNOS production, required for the conversion from an acute tachyzoite-driven to a persistent bradyzoite infection in tissue cysts and host survival.

## Data Availability Statement

The raw data supporting the conclusions of this article will be made available by the authors, without undue reservation.

## Ethics Statement

The animal study was reviewed and approved by Ministerium für Landwirtschaft, Energiewende, Umwelt und Ländliche Räume, Kiel, Germany and the Institutional Animal Care and Use Committee of Cincinnati Children's Hospital, Cincinnati, OH, USA.

## Author Contributions

DB, CK, JK, and JA contributed to the conception and design of the study. DB, FM, BO, MK, and MH-L performed the experiments and analyzed the data. DB and JK wrote the manuscript. All authors contributed to the article and approved the submitted version.

## Conflict of Interest

The authors declare that the research was conducted in the absence of any commercial or financial relationships that could be construed as a potential conflict of interest.
