# Supplementary material for: C5aR1 Activation Drives Early IFN-γ Production to Control Experimental Toxoplasma gondii Infection
Source: Front Immunol. 2020 Jul 8;11:1397. doi: 10.3389/fimmu.2020.01397 (PMC7362728; doi:10.3389/fimmu.2020.01397)
Supplement: Supplementary file 1 [file Data_Sheet_1.DOCX]

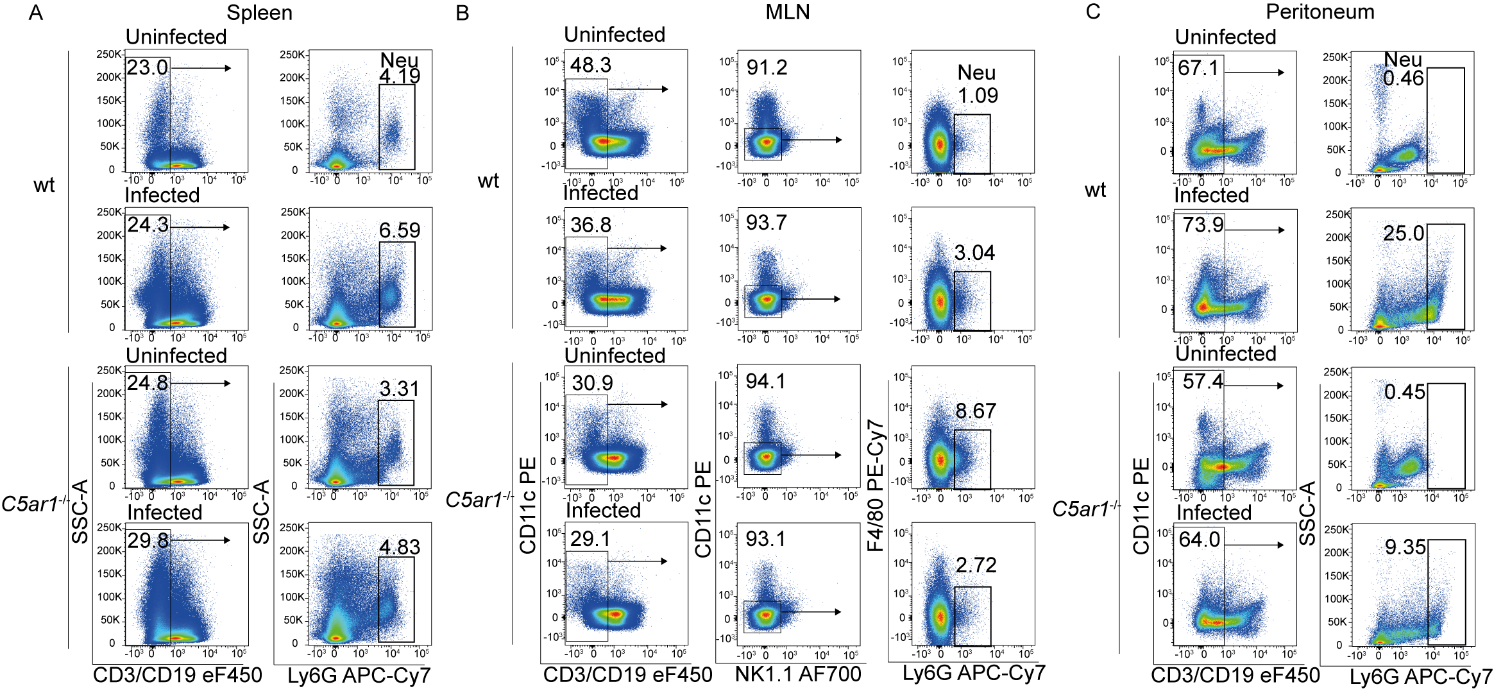
Supplementary Material

**Supplementary Figure 1. Gating strategy to identify neutrophils in the spleen, mesenteric lymph nodes (MLN) and peritoneum of wt and *C5ar1^-/-^* mice.** Representative dot plots depict neutrophil populations in (**A**) spleen (CD3/CD19^-^Ly6G^+^), (**B**) MLN CD3/CD19/NK1.1/F4/80^-^Ly6G^+^) and (**C**) peritoneal cavity (CD3/CD19^-^Ly6G^+^) of uninfected wt or *C5ar1^-/-^* mice and five days after *T. gondii* infection. Numbers represent frequencies of the parent gate.

**Supplementary Figure 2. Baseline gene expression in the brain and spleen of naïve wt and *C5ar1*^-/-^ mice.** (**A**) *Nos2, Il12a, Il12b, Il18,* and *Ifng* and *Il18* mRNA transcription was determined in the brain and (**B**) *Ifng* mRNA transcription was determined in the spleen of wt and *C5ar1*^-/-^ mice by RT-qPCR. The data show the relative mRNA transcripts in comparison to β-actin, n = 3-5/group. Values shown are the mean ± SEM, differences between groups were determined by unpaired t-test, *** *p* < 0.001.


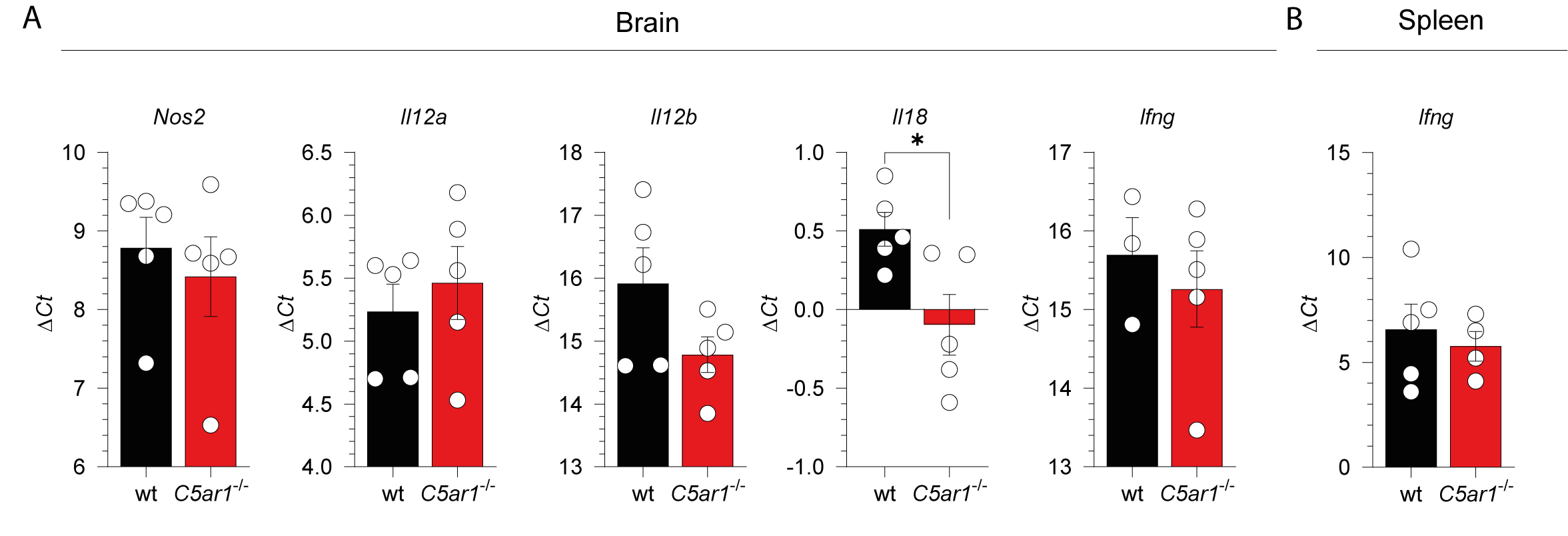


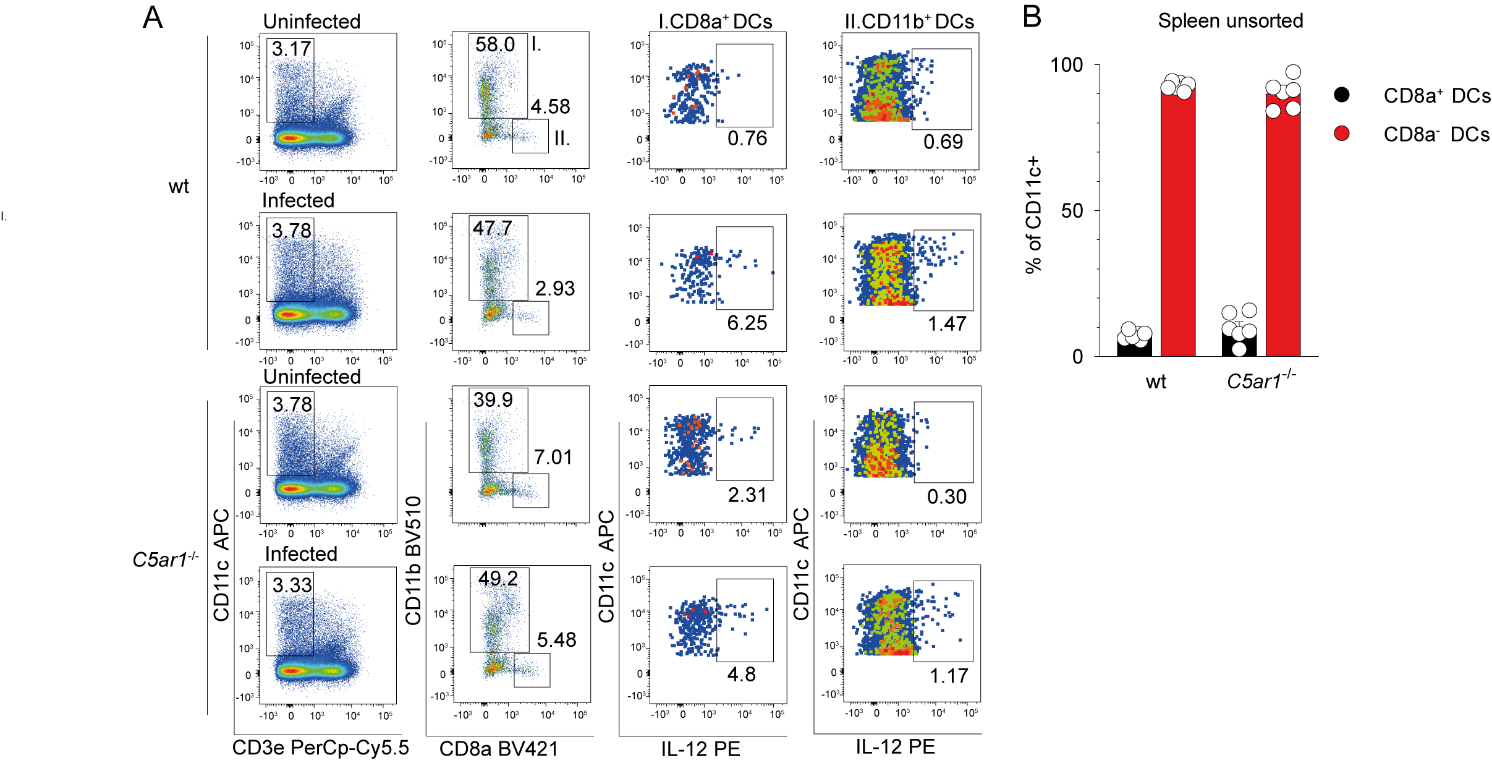


**Supplementary Figure 3. Gating strategy to identify IL-12p40 production in CD8^+^ and CD11b^+^ DCs in the spleen of wt and *C5ar1^-/-^* mice.** (**A**) Representative dot plots depicting intracellular IL-12p40 staining in spleen cells from uninfected wt or *C5ar1^-/-^* mice five days after *T. gondii* infection. CD11b DCs were identified as CD3^-^CD11c^+^CD11b^+^CD8^-^ (I) and CD8α^+^ DCs were identified as CD3^-^CD11c^+^CD11b^-^CD8a^+^ (II). Numbers represent frequencies of the parent gate. (**B**) Frequencies of CD8a^+^ (black) and CDD8a^-^ (red) cells within the population of CD11c^+^ DCs in the spleen of naïve wt and *C5ar1^-/-^* mice.


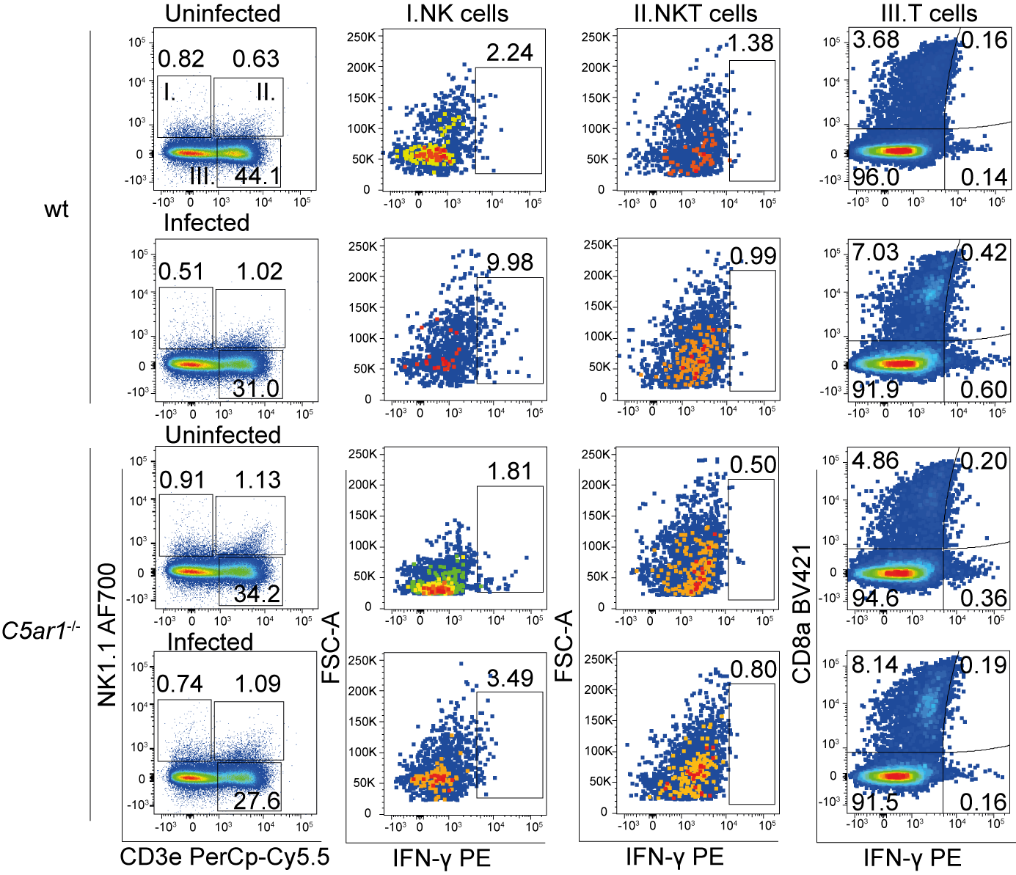


**Supplementary Figure 4. Gating strategy to identify IFN-γ-producing NK, NKT and T cells in the spleen of naïve wt and *C5ar1^-/-^* mice.** Representative dot plots depict intracellular IFN-γ production in (**I**) NK (CD3e^-^NK1.1^+^), (**II**) NKT (CD3e^+^NK1.1^+^) and (**III**) T cells (NK1.1^-^CD3e^+^CD8^+^ and NK1.1^-^CD3e^+^CD8^-^) in the spleen of uninfected wt or *C5ar1^-/-^* mice and five days after *T. gondii* infection. Numbers represent frequencies of the parent gate.
